# Nanosilicate-functionalized nanofibrous membrane facilitated periodontal regeneration potential by harnessing periodontal ligament cell-mediated osteogenesis and immunomodulation

**DOI:** 10.1186/s12951-023-01982-4

**Published:** 2023-07-13

**Authors:** Xiongcheng Xu, Ziqin Chen, Long Xiao, Yanmei Xu, Nianqi Xiao, Weiqiu Jin, Yuling Chen, Yanfen Li, Kai Luo

**Affiliations:** 1 Fujian Key Laboratory of Oral Diseases & Fujian Provincial Engineering Research Center of Oral Biomaterial & Stomatological Key laboratory of Fujian College and University, School and Hospital of Stomatology, Fujian Medical University, Fuzhou, People’s Republic of China; 2 Institute of Stomatology & Laboratory of Oral Tissue Engineering, School and Hospital of Stomatology, Fujian Medical University, Fuzhou, 350002 People’s Republic of China; 3 Nanjing Stomatological Hospital, Medical School of Nanjing University, Nanjing, 210008 People’s Republic of China; 4 School and Hospital of Stomatology, Fujian Medical University, Fuzhou, 350002 People’s Republic of China; 5 Nanjing Stomatological Hospital, Medical School of Nanjing University, Nanjing, 210008 People’s Republic of China

**Keywords:** Nanosilicate, Periodontal ligament, Osteogenesis, Immunomodulation, Periodontal regeneration

## Abstract

Although various new biomaterials have enriched the methods for periodontal regeneration, their efficacy is still controversial, and the regeneration of damaged support tissue in the periodontium remains challenging. Laponite (LAP) nanosilicate is a layered two-dimensional nanoscale, ultrathin nanomaterial with a unique structure and brilliant biocompatibility and bioactivity. This study aimed to investigate the effects of nanosilicate-incorporated PCL (PCL/LAP) nanofibrous membranes on periodontal ligament cells (PDLCs) in vitro and periodontal regeneration in vivo. A PCL/LAP nanofibrous membrane was fabricated by an electrospinning method. The characterization of PCL/LAP nanofibrous membrane were determined by scanning electron microscopy (SEM), energy dispersive spectrum of X-ray (EDS), inductively coupled plasma mass spectrometry (ICP-MS) and tensile test. The proliferation and osteogenic differentiation of PDLCs on the PCL/LAP nanofibrous membrane were evaluated. A PDLCs and macrophage coculture system was used to explore the immunomodulatory effects of the PCL/LAP nanofibrous membrane. PCL/LAP nanofibrous membrane was implanted into rat calvarial and periodontal defects, and the regenerative potential was evaluated by microcomputed topography (micro-CT) and histological analysis. The PCL/LAP nanofibrous membrane showed good biocompatibility and bioactivity. It enhanced the proliferation and osteogenic differentiation of PDLCs. The PCL/LAP nanofibrous membrane also stimulated anti-inflammatory and pro-remodeling N2 neutrophil formation, regulated inflammatory responses and induced M2 macrophage polarization by orchestrating the immunomodulatory effects of PDLCs. The PCL/LAP nanofibrous membrane promoted rat calvarial defect repair and periodontal regeneration in vivo. LAP nanosilicate-incorporated PCL membrane is capable of mediating osteogenesis and immunomodulation of PDLCs in vitro and accelerating periodontal regeneration in vivo. It could be a promising biomaterial for periodontal regeneration therapy.

## Introduction

Periodontitis affects the supporting tissues of the teeth, including causing alveolar bone resorption and loss of attachment, ultimately leads to teeth loss [1]. Periodontal therapy aims to achieve regeneration of lost/damaged periodontal tissue. Alveolar bone is recognized as the basis of structural and functional regeneration of periodontal tissue, which supports and stabilizes the cementum-periodontal ligament-alveolar bone complex [1]. Various grafts and barrier membranes have been used in clinical practice to provide and maintain a stable microenvironment for periodontal regeneration [2, 3]. However, periodontal regeneration is currently unpredictable in severe periodontal defects, partly due to the limited pro-regenerative effect of biomaterials for clinical applications.

Polycaprolactone (PCL) was previously approved by the U.S. Food and Drug Administration (FDA) for several medical applications. PCL is an absorbable synthetic polymer, and its degradation products would not lead to a significant enough decrease in the pH of the local microenvironment to compromise tissue regeneration [4]. Recently, PCL was applied in periodontal regeneration research [5, 6]. Farage et al. developed decellularized tissue engineered constructs to facilitate periodontal regeneration by constructing decellularized periodontal ligament cell (PDLC) sheets on electrospun PCL membranes [7, 8]. PCL nanofibrous membranes coated with polydopamine could mimic the extracellular environment for periodontal and bone tissue repair [9]. Pilipchuk et al. fabricated PCL scaffolds integrating a 3D-printed bone region with a micropatterned PCL thin film and found that the PCL scaffold had the ability to form periodontium-like complexes in vivo [10]. These studies indicated that PCL shows potential in periodontal regeneration, but it cannot directly regulate the regeneration process due to the limited pro-regenerative effects of pure PCL. Therefore, there is an urgent need to develop functional modifications that enrich the regenerative properties of PCL.

Laponite (LAP), a synthetic nanosilicate, is a layered two-dimensional nanoscale ultrathin nanomaterial [11]. It has been extensively explored for regenerative biomedical applications due to its unique structure and brilliant biocompatibility and bioactivity [11, 12]. Due to the special composition and disk-like shape, LAP exhibits a high surface area and dual-charge distribution with a permanent negative charge on the surface and a positive charge along the edge, which facilitates physical interactions with various biomolecules and biomaterials [12]. LAP degrades into bioactive ionic products, including Mg^2+^, Si(OH)_4_ and Li^+,^ in vivo, which is advantageous for tissue regeneration [13–15]. Our previous studies confirmed the brilliant pro-regeneration potential of LAP in the biofunctional modification of biomaterials, especially in the field of bone regeneration [16, 17]. Incorporation of LAP into PCL increased the mechanical properties and orchestrated osteoblasts and bone mesenchymal stem cells to directly induce osteogenesis and indirectly regulate angiogenesis and osteoclastogenesis, ultimately accelerating bone regeneration [16, 17]. However, it is unclear whether LAP-incorporated PCL promotes periodontal regeneration.

Periodontal ligament cells (PDLCs), which are derived from periodontal tissues, have been recognized as the main cells that participate in periodontal regeneration, including periodontal ligament, alveolar bone and cementum [18, 19]. Increasing evidences indicate that PDLCs also act as an immunomodulatory role by secreting cytokines to regulate regeneration process [20–22]. An ideal biomaterial for periodontal regeneration therapy should orchestrate PDLCs to directly participate in periodontal tissue formation and indirectly provide a immune microenvironment in favor of regeneration. In the present study, the LAP-incorporated PCL composite was applied to fabricate electrospun nanofibrous membranes and was cocultured with PDLCs in vitro to explore the biocompatibility and immunomodulatory effects. Subsequently, the PCL/LAP nanofibrous membranes were implanted into a rat calvarial and periodontal defect model. Both microcomputed tomography (micro-CT) and histological assessment were performed to evaluate its effect on periodontal regeneration.

## Experimental section

### Materials

Polycaprolactone (PCL) with an average molecular weight of 80000 g/mol, dexamethasone, ascorbic acid and β-glycerophosphate were purchased from Sigma-Aldrich, USA. Dulbecoo’s Modified Eagle Medium (DMEM) and Fetal Bovine Serum (FBS) were obtained from Hyclone Co. Ltd. Adipogenic differentiation medium and chondrogenic differentiation medium were obtained from Cyagen Biosciences Co. Ltd. FITC-labeled CD34, FITC-labeled CD45, FITC-labeled CD73, FITC-labeled CD90 were obtained from BD Biosciences Co. Ltd. Rhodamine phalloidin was obtained from Cytoskeleton Co. Ltd. 4’,6-diamidino-2-phenylindole (DAPI) solution, live/dead cell staining kit, BCIP/NBT reagent, RIPA lysis buffer, BCA protein assay kit and enhanced chemiluminescence reagents were obtained from Beyotime Co. Ltd. CCK-8 assay kit was obtained from Dojindo Co. Ltd. ALPL assay kit was obtained from Jiancheng Inc. TRIzol reagent was obtained from Life Technologies. PrimeScript RT reagent kit and SYBR green PCR mix were obtained from Takara Co. Ltd. Polyvinylidene difluoride (PVDF) membranes was obtained from Millipore Co. Ltd. Vimentin, cytokeratin, RUNX2, ALPL, COL1A1, GAPDH, iNOS, ARG1 antibodies, Alexa Flour-488 conjugated secondary antibody and IL-1β, IL-4, IL-6, IL-10, IFN-γ, TNF-α and VEGFA ELISA kit were obtained from Abcam Co. Ltd. Secondary antibody was obtained from Boster Co. Ltd. Hematoxylin and eosin (H&E) and Masson’s trichrome staining were obtained from Maixin Biotechnology.

### Preparation and characterization of PCL/LAP membranes

PCL and PCL/5wt%LAP (referred to as PCL/LAP) composites were fabricated by a solvent-exchange method as described in our previous study [17]. PCL and PCL/LAP nanofibrous membranes were fabricated by electrospinning. The PCL or PCL/LAP composites were dissolved in hexafluoroisopropanol (HFP) to obtain an 8 wt% solution. The electrospinning of PCL and PCL/LAP was carried out at 10 kV and a flow rate of 1 mL/h at room temperature under 30% humidity. The electrospun membranes were dried overnight in a vacuum to remove the residual solvent. The morphology of both electrospun membranes was observed by scanning electron microscopy (SEM) (Hitachi S4800 FEG, Japan). The elemental analyses was performed using energy-dispersive spectroscopy (EDS) (Hitachi S4800 FEG, Japan).

PCL/LAP nanofibrous membranes were immersed in normal saline solution with 6 cm^2^/mL extraction ratio (surface area/volume) at 37℃ for 3 days. The concentrations of bioactive ions (Si^4+^, Mg^2+^ and Li^+^) were determined by inductively coupled plasma mass spectrometry (ICP-MS, Agilent 7700ce, USA).

To evaluate the degradation characteristic, PCL and PCL/LAP nanofibrous membranes were cut into 10 mm×40 mm rectangle shapes and recorded their initial weight. Then they were incubated in PBS at 37 ℃. At each time point, the PBS was carefully removed, and the nanofibrous membranes were washed with distilled water. They were subsequently frozen and lyophilized. The weight of the degraded nanofibrous membranes were measured, and the percentage mass loss was calculated based on their initial weight before incubation.

The mechanical properties of the both membranes (10 × 50 mm^2^) were detected by the universal testing machine (CMT-51, China). The stress-strain curves were obtained, and the tensile strength, strain at fracture and Young’s modulus were subsequently statistically analyzed. Both the PCL and PCL/LAP membranes were sterilized by ethylene oxide for the following experiments.

### PDLCs culture and identification

Ethical approval for the collection of PDLCs was attained through the School and Hospital of Stomatology, Fujian Medical University. Primary hPDLCs were harvested from healthy human PDL tissue as previously described [23] and cultured in DMEM with 10% FBS. PDLCs within passages 2 and 6 were used in the following studies.

The surface markers of PDLCs were analyzed by flow cytometry. PDLCs were digested with 0.25% trypsin and collected suspension to incubate with FITC-labeled CD34, FITC-labeled CD45, FITC-labeled CD73 and FITC-labeled CD90, respectively, then analyzed by flow cytometer (BD Biosciences, USA). For immunofluorescent staining, PDLCs were seeded on 12-well plates for 1 day and then fixed, permeabilized and blocked to be stained with anti-vimentin (1:200) or anti-cytokeratin (1:500) antibody, respectively. Then Alexa Flour-488 conjugated secondary antibody (1:400) was added and nuclei were stained with DAPI. The stained cells were imaged using fluorescence microscopy (Zeiss, Germany). Further, PDLCs were seeded on 24-well plates to confirm the capacity for multipotent differentiation. To evaluate osteogenic differentiation potential, PDLCs were cultured with osteogenic induction medium containing 10 nM dexamethasone, 50 µM ascorbic acid and 10 mM β-glycerophosphate for 14 days. Then the PDLCs were fixed and stained with Alizarin red S (ARS) (pH 4.2) solution to observe the formed calcium nodules under a microscope. To evaluate adipogenic differentiation potential, PDLCs were cultured in adipogenic differentiation medium for 14 days and fixed to be stained with Oil red O solution to observe lipid droplets. To explore chondrogenic differentiation potential, PDLCs were cultured in 15 mL sterile centrifuge tubes and incubated in chondrogenic differentiation medium for 21 days. The formed pellets were fixed and embedded. Sections were obtained to be stained with 0.05% alcian blue.

### Cytoskeleton staining

PDLCs were seeded on PCL and PCL/LAP nanofibrous membranes for 1 day and then fixed, permeabilized, and blocked to be stained with rhodamine phalloidin (1:200) and DAPI solution. The stained cells were imaged using fluorescence microscopy (Zeiss, Germany).

### Live/dead cell staining

PDLCs were seeded on PCL and PCL/LAP nanofibrous membranes for 1 day and incubated with propidium iodide (PI) for dead cells (red) and calcein AM for live cells (green) for 15 min at 37 °C. Finally, the stained PDLCs on both membranes were imaged using fluorescence microscopy. The cell viability percentage was analyzed by dividing the number of live cells by the total number of cells.

### Cell adhesion and proliferation

A CCK-8 assay was used to evaluate PDLCs proliferation. PDLCs at each time point were determined after incubation with 10% CCK8 solution, and the optical density (OD) value at 450 nm was measured by using an iMark microplate reader (Bio-Rad, USA).

#### ALPL and mineral nodule formation assays

Alkaline Phosphatase (ALPL) staining was performed using BCIP/NBT reagent following osteogenic induction for 7 days. For ALPL activity, PDLCs on both nanofibrous membranes were lysed with RIPA lysis buffer and assessed using an ALPL assay kit and a BCA protein assay kit. ALPL activity was normalized to the total protein amount in the cell lysate. For mineral nodule formation analysis, PDLCs were cultured on both membranes following osteogenic induction for 14 days, stained with 40 mM ARS and quantified by eluting with 10% cetylpyridinium chloride in 10 mM sodium phosphate (pH 7.0).

### Quantitative real-time polymerase chain reaction (PCR)

RNA was extracted from PDLCs using TRIzol reagent and transcribed using a PrimeScript RT reagent kit. Gene expression was evaluated using a LightCycler 480 real-time PCR system (Roche Diagnostics, Germany) with SYBR green PCR mix. The primers (Table 1) were synthesized by Sangon Biotech (Shanghai, China).

**Table 1:** Primer sequences used in quantitative real-time PCR

Gene	Primers
*GAPDH (homo)*	Forward: 5′-ACCCACTCCTCCACCTTTGAC-3′ Reverse: 5′-TCCACCACCCTGTTGCTGTAG-3′
*RUNX2 (homo)*	Forward: 5′-ACCAGCAGCACTCCATATCTCTAC-3′ Reverse: 5′-CTTCCATCAGCGTCAACACCATC-3′
*ALPL (homo)*	Forward: 5′-ACTGGTACTCAGACAACGAGAT-3′ Reverse: 5′-ACGTCAATGTCCCTGATGTTATG-3′
*COL1A1 (homo)*	Forward: 5′-TCGGAGGAGAGTCAGGAAGG-3′ Reverse: 5′-TCAGCAACACAGTTACACAAGG-3′
*GAPDH (mus)*	Forward: 5’-AGGTGGTGAAGCAGGCATC − 3’ Reverse: 5’-AAGGTGGAAGAGTGGGAGTTG − 3’
*IL-1β (mus)*	Forward: 5’-CCAAGCAATACCCAAAGAAGAAGATG-3’ Reverse: 5’-TTATGTCCTGACCACTGTTGTTTCC-3’
*IL-6 (mus)*	Forward: 5’-GAAACCGCTATGAAGTTCCTCTCT-3’ Reverse: 5’-GTATCCTCTGTGAAGTCTCCTCTCC-3’
*IL-10 (mus)*	Forward: 5’-CCCCAGCCGCTTCATCCC-3’ Reverse: 5’-ACAAACAATACACCATTCCCAGAGG-3’
*TNF-α (mus)*	Forward: 5’-AAGGGAGAGTGGTCAGGTTGC-3’ Reverse: 5’-TGGAAAGGTCTGAAGGTAGGAAGG-3’
*iNOS (mus)*	Forward: 5’-GCACCACCCTCCTCGTTCAG-3’ Reverse: 5’-CCACAACTCGCTCCAAGATTCC-3’
*ARG1 (mus)*	Forward: 5’- GCCTTTGTTGATGTCCCTAATG-3’ Reverse: 5’- GCACCACACTGACTCTTCC-3’

### Western blot (WB)

After 7 days of osteogenic induction, proteins of PDLCs on both membranes were extracted using RIPA lysis buffer. Proteins were fractionated by gel electrophoresis and transferred onto PVDF membranes. The PVDF membranes were blocked and incubated with the primary antibodies RUNX2 (1:1000), ALPL (1:1000), COL1A1 (1:1000) and GAPDH (1:1000). Then, the PVDF membranes were incubated with a secondary antibody (1:2000). The protein expression levels were detected using enhanced chemiluminescence reagents and analyzed using ImageJ software.

### Neutrophils treated with PDLCs-conditioned medium

PDLCs were cocultured with both nanofibrous membranes with 1 mL culture medium in 24-well plates for 7 days. The supernatants were collected, centrifuged and filtered through 0.22-µm filters. The filtrate was mixed with DMEM containing 10% FBS to obtain conditioned medium (referred to as PDLCs-PCL-CM and PDLCs-PCL/LAP-CM). The supernatants of PDLCs without membranes were used as controls (PDLCs-CM). The HL-60 cells were incubated with 1.25% DMSO to induce neutrophils and then cultured in the above conditioned medium. The neutrophil shapes were imaged using inverted microscopy. The expression levels of inflammatory genes of the neutrophils were assessed by using quantitative real-time PCR (as described in the above) on Day 1. The levels of IL-4, IL-6, IL-10, IFN-γ, TNF-α and VEGFA in the supernatants were assessed using ELISA on Day 1.

### Macrophages treated with PDLCs-conditioned medium

The condition medium was obtained as described in the above. The RAW 264.7 macrophages were cultured in the above conditioned medium. The macrophage shapes were imaged using inverted microscopy. The expression levels of inflammatory genes of the macrophages were assessed by using quantitative real-time PCR (as described in the above) on Day 3. The supernatants of macrophages treated with conditioned medium were collected after 3 days of culturing. The levels of IL-1β, IL-6, IL-10 and TNF-α in the supernatants were assessed using ELISA.

### Immunofluorescent staining

Marcophages were fixed, permeabilized and blocked at Day 3. The primary antibody against iNOS (1:1000) or ARG1 (1:1000) was incubated onto the cells for 2 h. Alexa Flour-488 conjugated secondary antibody (1:400) was added. Then, the actin cytoskeleton was stained with rhodamine phalloidin (1:200), and nuclei were stained with DAPI. The stained cells were imaged using fluorescence microscopy.

### Rat calvarial defect surgical procedure

The study protocol was approved by the Animal Care and Use Committee of Fujian Medical University. All rats were anesthetized *via* intraperitoneal injection of 40 mg/kg ketamine. The calvarial defect model was prepared in 4-week-old rat calvarial. A round bur was used to prepare a critical-sized calvarial defect with a 6 mm diameter. 15 rats were split into three groups: the PCL group with PCL nanofibrous membrane placement, the PCL/LAP group with PCL/LAP nanofibrous membrane placement and the blank group without any implant as a negative control. 5 rats were included in each group. Eight weeks later, all rats were sacrificed to collect calvarias.

### Rat periodontal defect surgical procedure

The study protocol was approved by the Animal Care and Use Committee of Fujian Medical University. All rats were anesthetized via intraperitoneal injection of 40 mg/kg ketamine. The periodontal defect model was prepared in 4-week-old rat mandibles as described in a previous study [24]. A round bur was used to prepare periodontal defects with 2 mm height × 3 mm width × 1 mm depth around the first molar. The periodontium was removed to expose the root surface. 15 rats were split into three groups: the PCL group with PCL nanofibrous membrane placement, the PCL/LAP group with PCL/LAP nanofibrous membrane placement and the blank group without any implant as a negative control. 5 rats were included in each group. Four weeks later, all rats were sacrificed to collect mandibles.

### Microcomputed tomography (micro-CT) analysis

The fixed calvarias or mandibles samples were scanned by a micro-CT scanner (SCANCO µCT50, Switzerland). Three-dimensional images were obtained and analyzed using Mimics software (Mimics 17.0, Materialise, Leuven, Belgium). For the periodontal defect model, cross-sections of micro-CT reconstructed images were near apical third area. Bone volume per total volume ratio (BV/TV) and bone mineral density (BMD) were evaluated.

### Histologic staining and analysis

The calvarias and mandibles were decalcified in 10% ethylenediaminetetraacetic acid (EDTA), dehydrated and embedded. Consecutive sections were obtained from the defect area and stained using H&E and Masson’s trichrome staining. For the periodontal defect model, the sections were near apical third area. The new bone areas and new attachment formation rate were assessed using ImageJ software.

### Statistical analysis

Data are presented as the mean ± standard deviation. The t test or one-factor analysis of variance followed by Tukey’s HSD post hoc test was used to evaluate the differences among groups. Significance was accepted at P < 0.05 for all tests.

## Results

### Characterization of PCL/LAP nanofibrous membranes

**Fig. 1:**
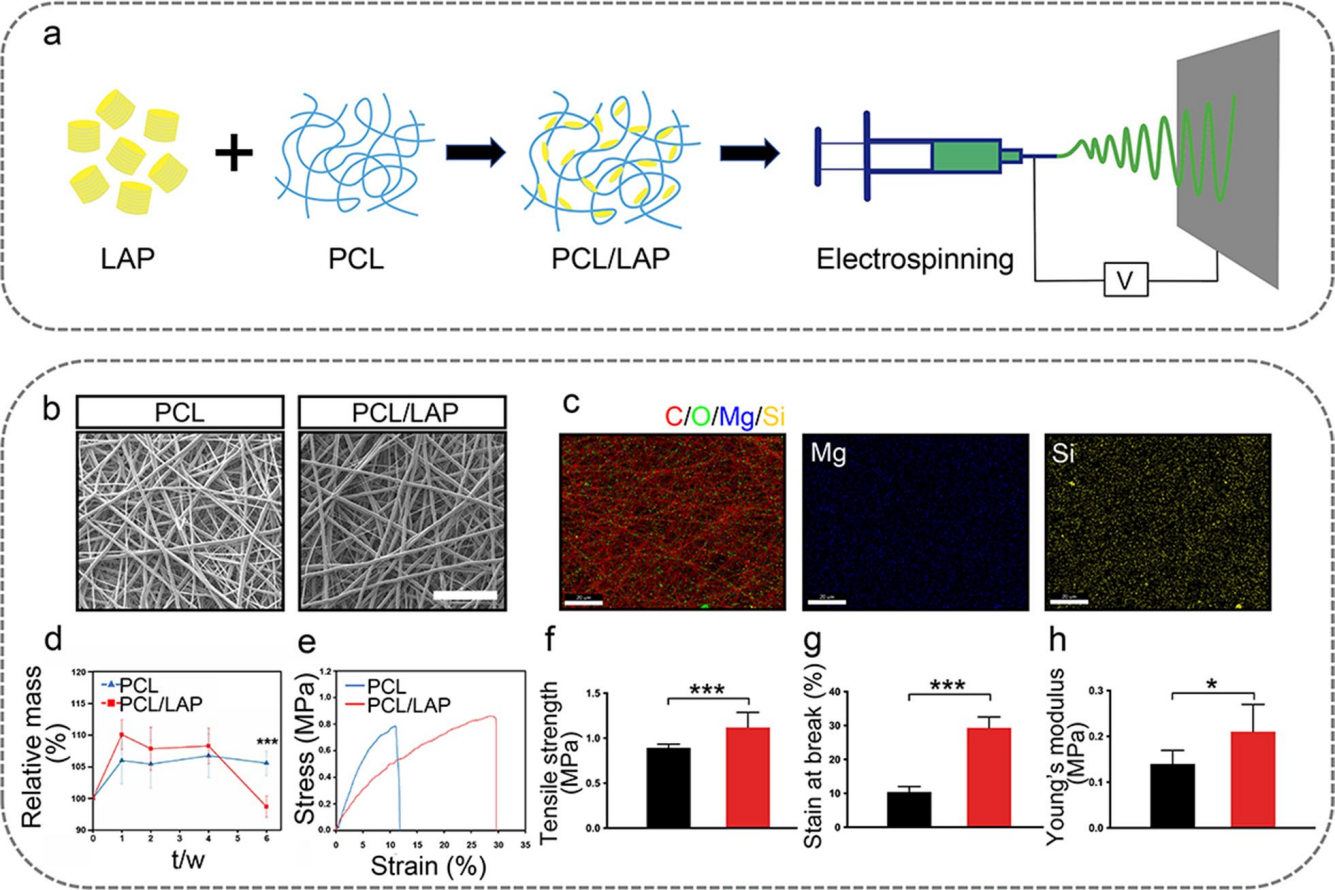
Characterization of PCL/LAP nanofibrous membranes. **a** Schematic diagram of the fabrication of PCL/LAP nanofibrous membrane. **b** SEM of PCL and PCL/LAP nanofibrous membranes. Scale bar = 20 μm. **c** EDS mapping analysis of PCL/LAP. Scale bar = 20 μm. **d** Degradation characteristics of PCL and PCL/LAP nanofibrous membranes. **e** The representative stress-strain curves of PCL and PCL/LAP nanofibrous membranes. **f** Tensile strength. **g** Strain at break. **h** Young’s modulus. Scale bar = 100 μm. *P ≤ 0.05, **P ≤ 0.01, ***P ≤ 0.001

PCL and PCL/LAP nanofibrous membranes were fabricated by using the electrospinning technique (Fig. 1a). Figure 1b shows their morphological appearances in SEM images, which featured randomly oriented filamentous architecture. The elemental analyses was performed using EDS. The presence of Mg and Si confirmed successful fabrication of nanofibrous membranes with LAP incorporation (Fig. 1c). Further, the ions released from PCL/LAP nanofibrous membrane were evaluated, and bioactive ions, including Mg^2+^, Si^4+^ and Li^+^, could be detected after 3 days of immersion. The result indicated that the concentration of released Mg^2+^ was about 181.47 ± 9.83 µg/mL, Si^4 +^ was about 396.87 ± 14.45 µg/mL and Li ^+^ was about 18.37 ± 1.00 µg/mL.

The degradation characteristics of both nanofibrous membranes were investigated in PBS at 37 °C (Fig. 1d). The mass of both nanofibrous membranes increased after 1 week, which might be attributed to their absorption capacities. The mass of PCL/LAP nanofibrous membrane significantly decreased after 6 weeks, but PCL nanofibrous membrane did not obviously change. This result indicated both PCL and PCL/LAP nanofibrous membranes could maintain integrity of membrane before 6 weeks, and addition of LAP would slightly promote degradation of PCL nanofibrous membrane.

Tensile tests were conducted to evaluate mechanical property of PCL/LAP nanofibrous membrane. The representative stress-strain curves were presented in Fig. 1e. Further, the characteristic parameters such as tensile strength, young’s modulus and strain at break calculated from these curves (Fig. 1f-h). These results showed higher levels of tensile strength, young’s modulus and strain at break of PCL/LAP nanofibrous membrane, which indicated that LAP incorporation would significantly increase mechanical property of PCL/LAP nanofibrous membrane.

### Biocompatibility of the PCL/LAP nanofibrous membrane on PDLCs

**Fig. 2:**
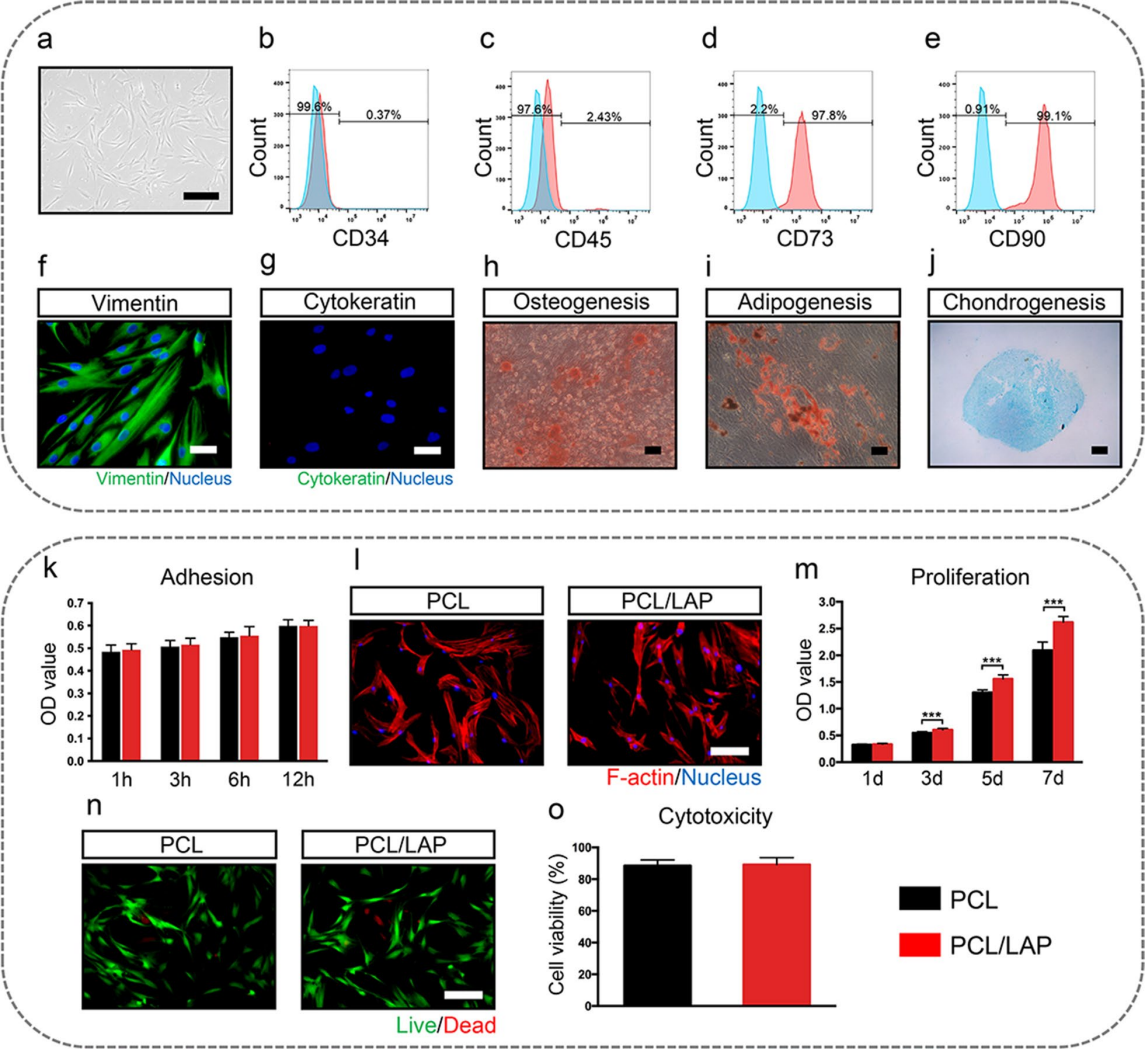
Biocompatibility of PCL/LAP nanofibrous membranes. **a** PDLCs cultured from human PDL tissue. **b-e** Flow cytometry analysis of CD34, CD45, CD73 and CD90 in PDLCs. **f-g** Immunofluorecent staining of vimentin and cytokeratin in PDLCs. **h** ARS staining. **i** Oil red O staining. **j** Alcian blue staining. **k** Adhesion of PDLCs on PCL and PCL/LAP nanofibrous membranes. **l** Cytoskeleton staining of PDLCs on PCL and PCL/LAP nanofibrous membrane. **m** Proliferation of PDLCs on PCL and PCL/LAP nanofibrous membranes. **n** Live/dead staining and analysis of PDLCs on PCL and PCL/LAP nanofibrous membranes. Scale bar = 100 μm. *P ≤ 0.05, **P ≤ 0.01, ***P ≤ 0.001

PDLCs were cultured from human PDL and displayed long spindle shapes (Fig. 2a). Flow cytometry analysis indicated that PDLCs were positive for mesenchymal stem cell markers (CD73 and CD90) (Fig. 2d, e), and were negative for hematopoietic cell marker CD34 and leukocyte maker CD45 (Fig. 2b, c). The immunofluorecent staining demonstrated the PDLCs expressed fibroblast marker vimentin and no cytokeratin-positive cell could be observed (Fig. 2f, g). PDLCs were successfully induced osteogenic, adipogenic or chondrogenic differentiation, which confirmed the capacity of multipotent differentiation (Fig. 2h, j).

The adhesion of PDLCs on both nanofibrous membranes was similar and showed no significant difference (Fig. 2k). PDLCs cultured on the surface of both membranes showed spreading shapes (Fig. 2l). Live/dead staining results demonstrated that the PCL/LAP nanofibrous membranes did not affect the viability of PDLCs (Fig. 2n, o). Moreover, PCL/LAP nanofibrous membranes promoted the proliferation of PDLCs after 3 days of culturing (Fig. 2m). This result indicated that the PCL/LAP nanofibrous membrane in the present study possessed good biocompatibility.

### PCL/LAP nanofibrous membrane enhances osteogenic differentiation of PDLCs

**Fig. 3:**
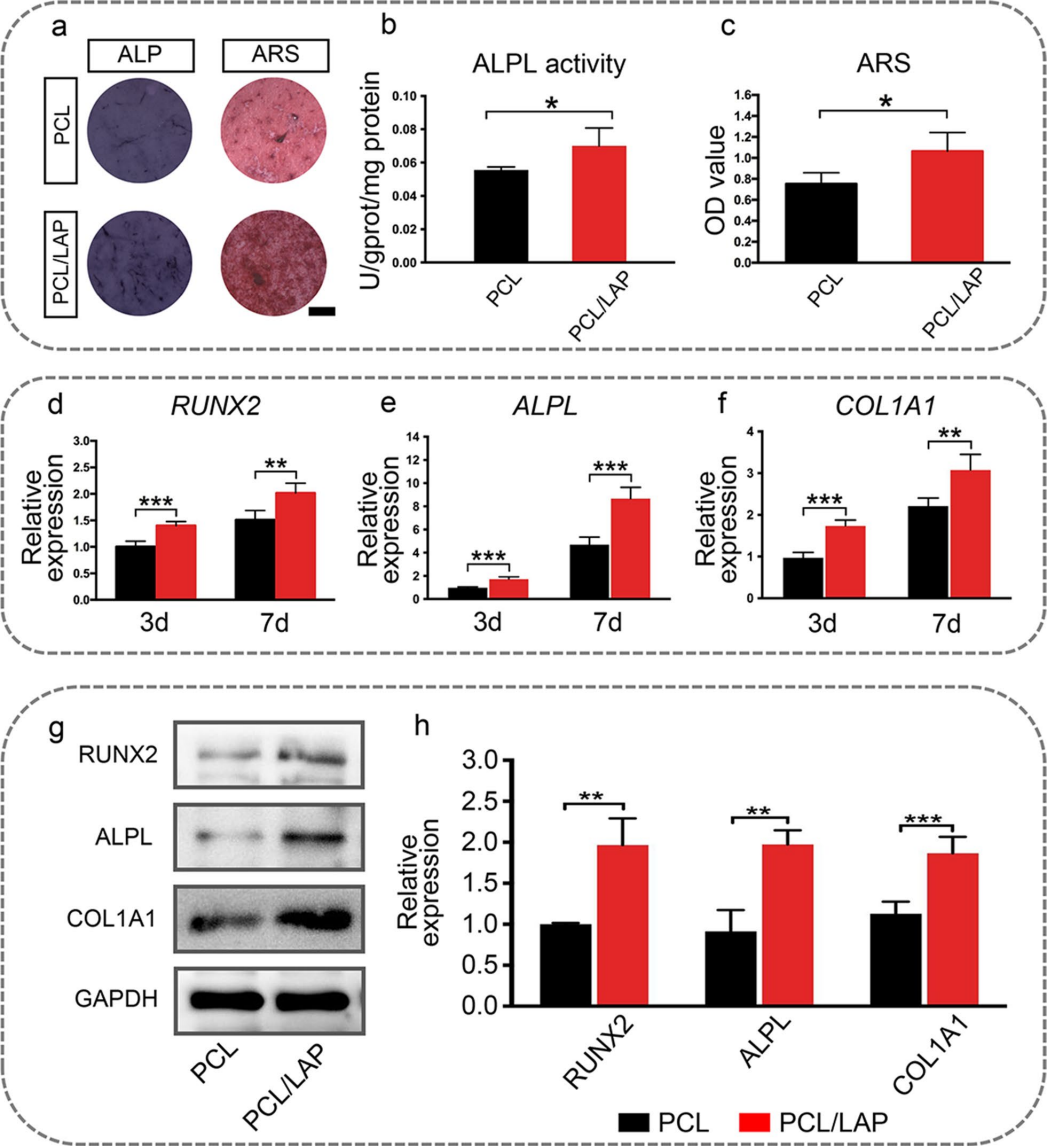
PCL/LAP nanofibrous membrane enhances osteogenic differentiation of PDLCs. The PCL/LAP nanofibrous membrane enhances the osteogenic differentiation of PDLCs. **a** ALPL and ARS staining of PDLCs on PCL and PCL/LAP nanofibrous membranes. **b** ALPL activity of PDLCs on PCL and PCL/LAP nanofibrous membranes. **c** Quantification of ARS staining of PDLCs on PCL and PCL/LAP nanofibrous membranes. **d-f** Expression levels of osteogenic differentiation genes (*RUNX2*, *ALPL* and *COL1A1*) of PDLCs on PCL and PCL/LAP nanofibrous membranes. **g-h** Western blot analysis of osteogenic differentiation proteins (RUNX2, ALPL and COL1A1) of PDLCs on PCL and PCL/LAP nanofibrous membranes. Scale bar = 1 mm. *P ≤ 0.05; **P ≤ 0.01; ***P ≤ 0.001

As shown in Fig. 3a, b, PDLCs cultured on the PCL/LAP nanofibrous membrane expressed higher levels of ALPL than PDLCs cultured on the PCL nanofibrous membrane. More mineral nodule formation could be found on the PCL/LAP nanofibrous membrane after ARS staining (Fig. 3a and c). Furthermore, the expression levels of osteogenic differentiation genes and proteins (RUNX2, ALPL and COL1A1) were evaluated using quantitative real-time PCR and WB analysis (Fig. 3d–h). The PCL/LAP nanofibrous membrane significantly upregulated the expression of RUNX2, ALPL and COL1A1 in PDLCs at the mRNA and protein levels. These results suggest that the PCL/LAP nanofibrous membrane enhances the osteogenic differentiation of PDLCs.

### Conditioned medium from PDLCs cultured on PCL/LAP nanofibrous membranes potential induces anti-inflammatory and pro-remodeling N2 neutrophils polarization

**Fig. 4:**
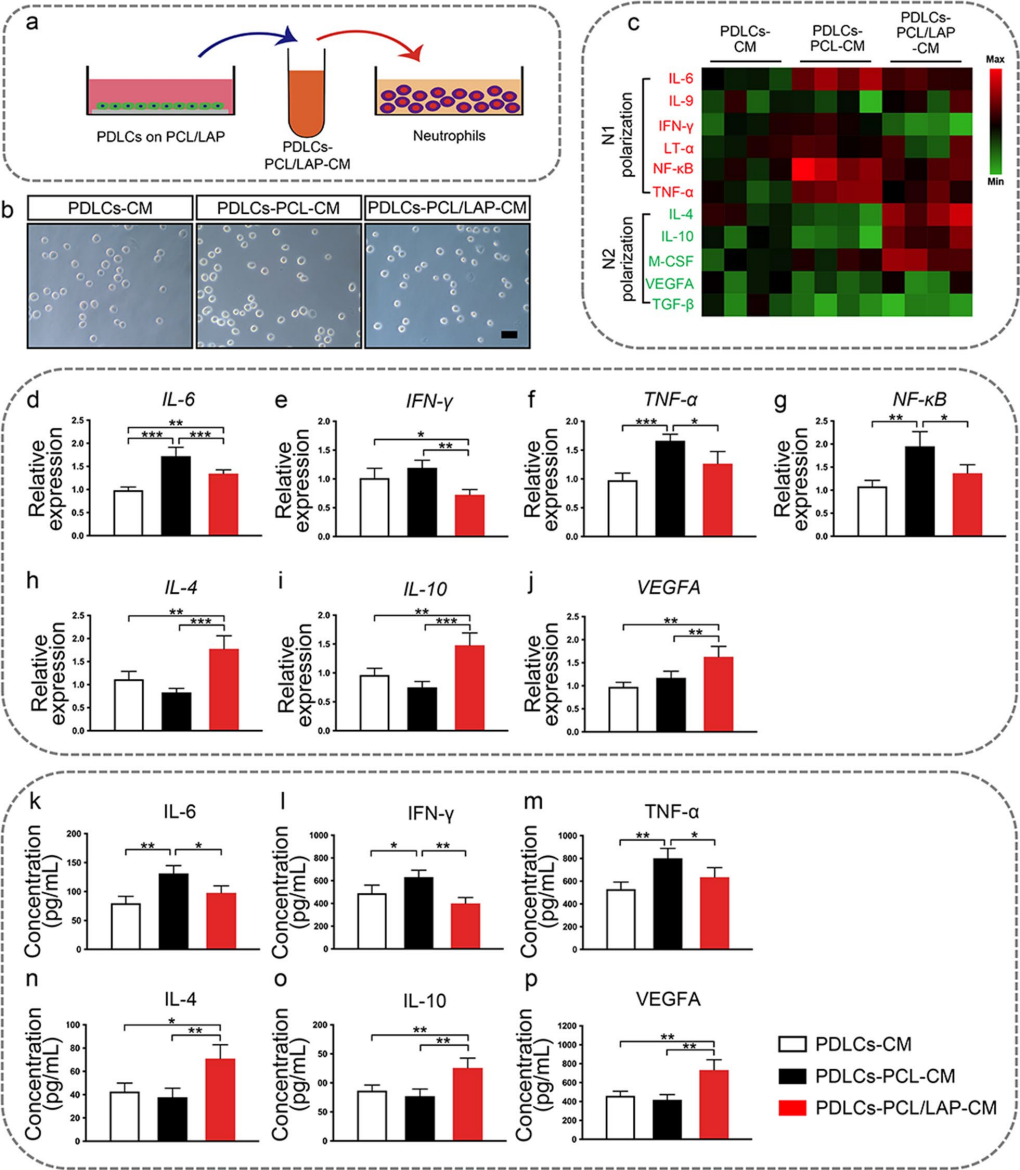
Conditioned medium from PDLCs cultured on PCL/LAP nanofibrous membranes potential induces anti-inflammatory and pro-remodeling N2 neutrophils polarization. **a** Schematic illustration of neutrophils treated by conditioned medium from PDLCs. **b** Representative appearances of neutrophils treated with different condition medium. **c** Heat maps of gene expression levels in neutrophils treated with condition medium from PDLCs cultured on PCL/LAP nanofibrous membranes. **d-g** Pro-inflammatory gene *(IL-6, IFN-γ, TNF-α* and *NF-κB*) levels of neutrophils. **h-i** Anti-inflammatory gene (*IL-4* and *IL-10*) expression of neutrophils. **j** Pro-remodeling gene (*VEGFA*) expression of neutrophils. **k-m** Pro-inflammatory cytokines (IL-6, IFN-γ and TNF-α) levels of neutrophils. **n-o** Anti-inflammatory cytokines (IL-4 and IL-10) levels of neutrophils. **j** Pro-remodeling cytokine (VEGFA) level of neutrophils. Scale bar = 30 μm. *P ≤ 0.05; **P ≤ 0.01; ***P ≤ 0.001

Neutrophils were treated with conditioned medium from PDLCs cultured on PCL/LAP nanofibrous membranes (Fig. 4a). As shown in Fig. 4b, neutrophils presented round shapes after different conditioned medium stimulation. Inflammatory mRNA expression of distinct neutrophil subpopulations were evaluated (Fig. 4c). Neutrophils treated with condition medium from PDLCs cultured on PCL/LAP nanofibrous membranes expressed lower canonical pro-inflammatory genes (*IL-6, IFN-γ, TNF-α* and *NF-κB*) (Fig. 4d–g), and higher anti-inflammatory genes (*IL-4* and *IL-10*) (Fig. 4h, i) and pro-remodeling gene (*VEGFA*) (Fig. 4j). The decreased pro-inflammatory mRNA levels and increased anti-inflammatory and pro-remodeling mRNA levels of neutrophils are consistent with an alternative N2 polarization state. Further, the cytokines in the supernatants were detected by ELISA. The results demonstrated neutrophils treated with conditioned medium from PDLCs cultured on PCL/LAP nanofibrous membranes secreted lower pro-inflammatory cytokines (IL-6, IFN-γ and TNF-α), and higher anti-inflammatory cytokines (IL-4 and IL-10) and pro-remodeling cytokine (VEGFA) (Fig. 4k–p). This result implied PCL/LAP nanofibrous membrane might potential induce anti-inflammatory and pro-remodeling N2 neutrophils polarization.

### Conditioned medium from PDLCs cultured on PCL/LAP nanofibrous membranes suppresses macrophage inflammation

**Fig. 5:**
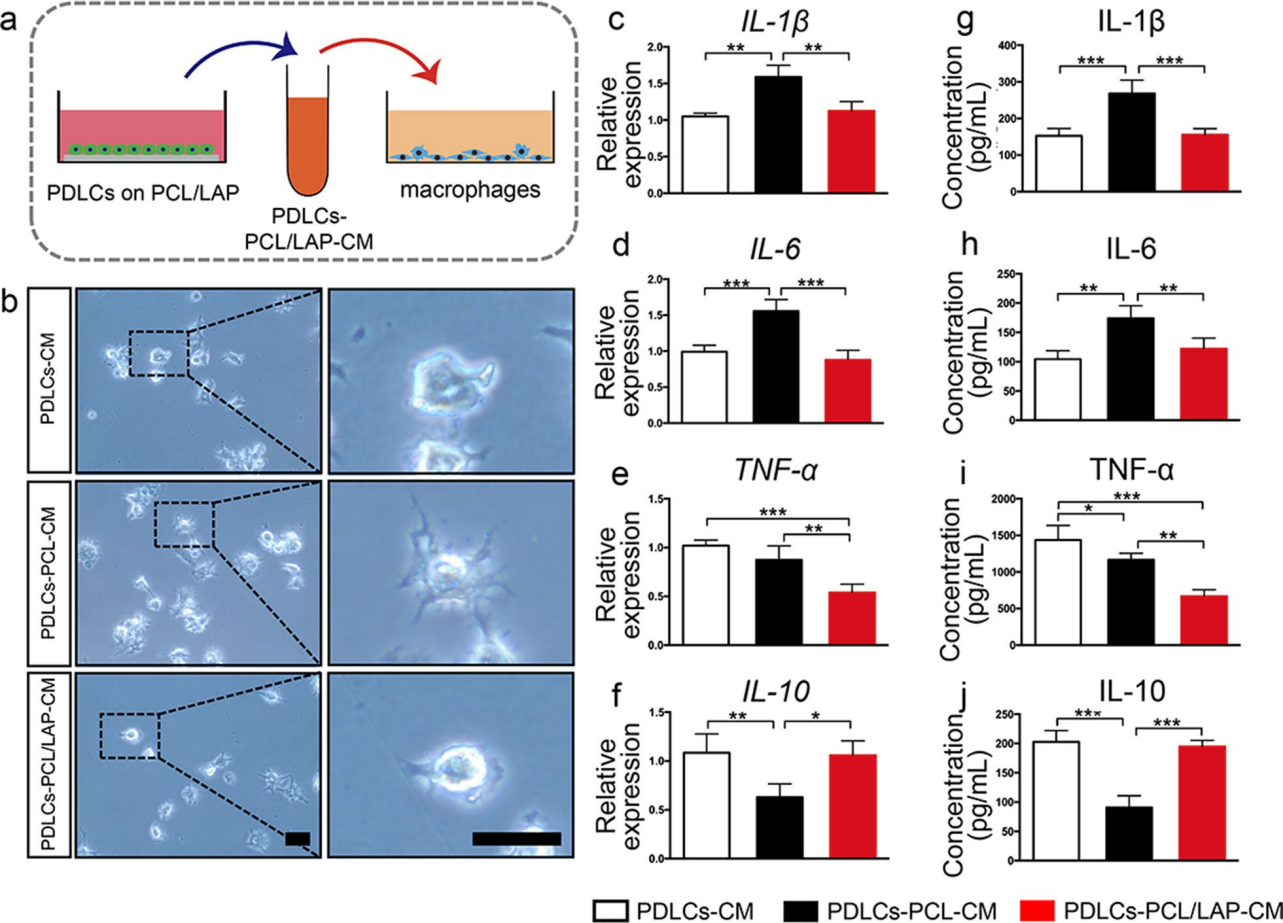
Conditioned medium from PDLCs cultured on PCL/LAP nanofibrous membranes inhibits macrophage inflammatory responses. **a** Schematic illustration of macrophages treated by conditioned medium from PDLCs. **b** Representative images of macrophages treated with conditioned medium from PDLCs cultured on PCL/LAP nanofibrous membranes. **c-f** Inflammatory gene (*IL-1β, IL-6, TNF-α* and *IL-10*) expression of macrophages treated with conditioned medium from PDLCs cultured on PCL/LAP membranes. **g**–**j** Inflammatory cytokines (IL-1β, IL-6, TNF-α and IL-10) of macrophages treated with conditioned medium from PDLCs cultured on PCL/LAP membranes. Scale bar = 30 μm. *P ≤ 0.05; **P ≤ 0.01; ***P ≤ 0.001

To evaluate whether PDLCs in response to PCL/LAP nanofibrous membranes exert immunomodulatory effects, the supernatants of PDLCs cultured on PCL (PDLCs-PCL-CM) and PCL/LAP (PDLCs-PCL/LAP-CM) nanofibrous membranes were collected and treated with macrophages (Fig. 5a). The supernatant of cultured PDLCs (PDLCs-CM) was also added to macrophages as a control. Figure 5b demonstrates that macrophages treated with PDLCs-PCL/LAP-CM showed fewer pseudopodia than those treated with PDLCs-PCL-CM, which indicated that PDLCs-PCL/LAP-CM could alleviate the morphological activation of macrophages. Both quantitative real-time PCR and ELISA were used to assess the inflammatory cytokine expression of macrophages treated with conditioned medium. PDLCs-PCL/LAP-CM significantly downregulated proinflammatory cytokines (IL-1β, IL-6 and TNF-α) at the mRNA (Fig. 5c-e) and protein (Fig. 5g-i) levels. Additionally, the anti-inflammatory cytokine (IL-10) levels of macrophages in response to PDLCs-PCL/LAP-CM increased accordingly (Fig. 5f and j).

**Fig. 6:**
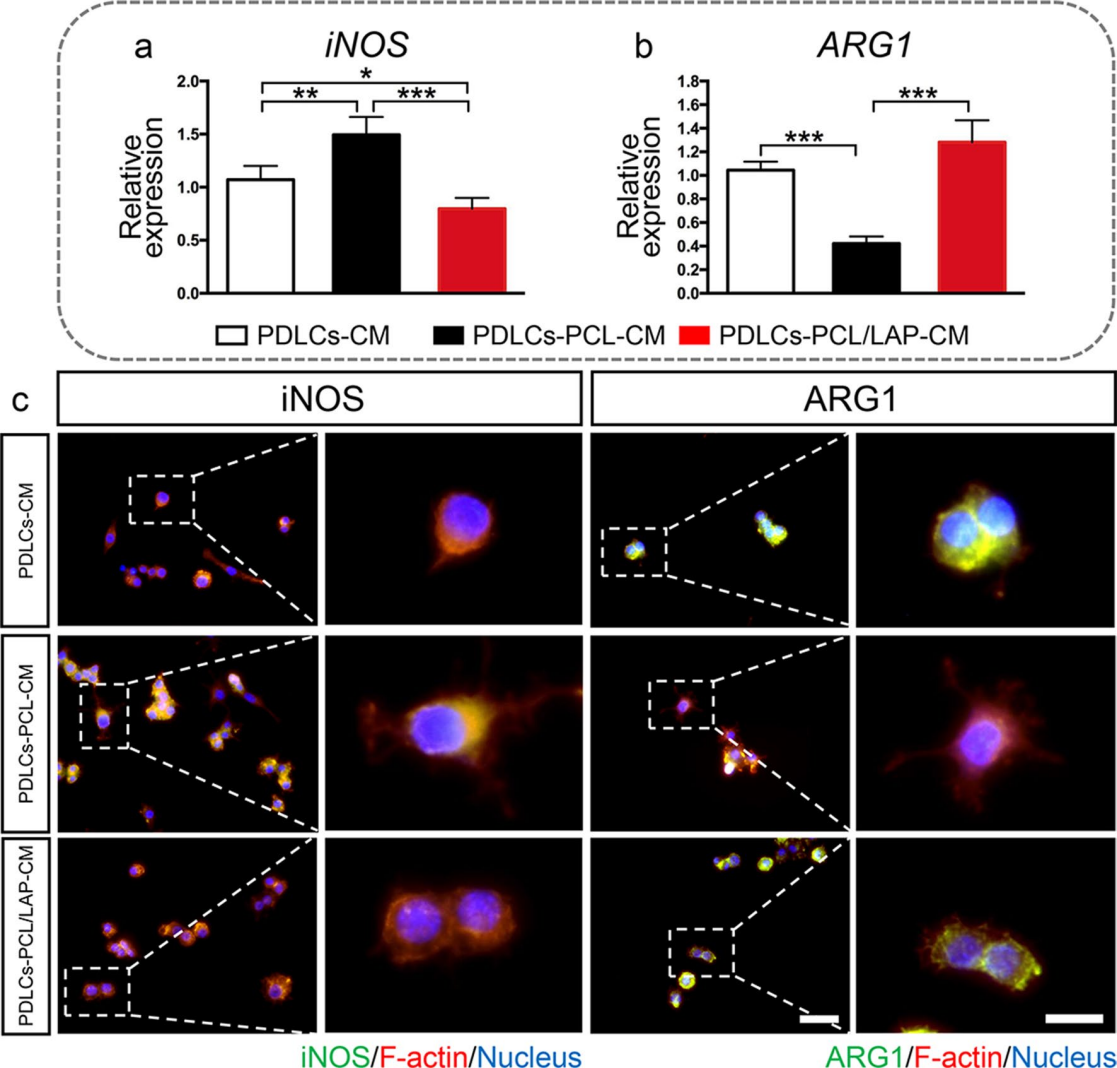
Conditioned medium from PDLCs cultured on PCL/LAP nanofibrous membranes regulates macrophage polarization. **a-b** Decreased M1 polarization marker (*iNOS*) gene and increased M2 polarization marker (*ARG1*) gene levels of macrophages treated with condition medium from PDLCs cultured on PCL/LAP nanofibrous membrane. **c** Representative images of immunofluorescent staining iNOS levels and ARG1 levels in macrophages treated with conditioned medium from PDLCs cultured on PCL/LAP nanofibrous membranes. Scale bar = 50 μm. *P ≤ 0.05; **P ≤ 0.01; ***P ≤ 0.001

The polarization of macrophages in response to each conditioned medium was evaluated. Figure 6a-b indicates that PDLC-PCL/LAP-CM inhibited the expression of the M1 macrophage marker (iNOS) gene and promoted the M2 macrophage marker (ARG1) level. Both polarization markers were assessed by using immunofluorescent staining. Lower iNOS levels and higher ARG1 levels were found in macrophages treated with PDLCs-PCL/LAP-CM in comparison to PDLCs-PCL-CM (Fig. 6c).

The results suggested that PDLCs cultured on the PCL/LAP membrane might manipulate immunomodulation by indirectly regulating the inflammatory responses of macrophages.

### PCL/LAP nanofibrous membrane accelerates rat calvarial bone formation

**Fig. 7:**
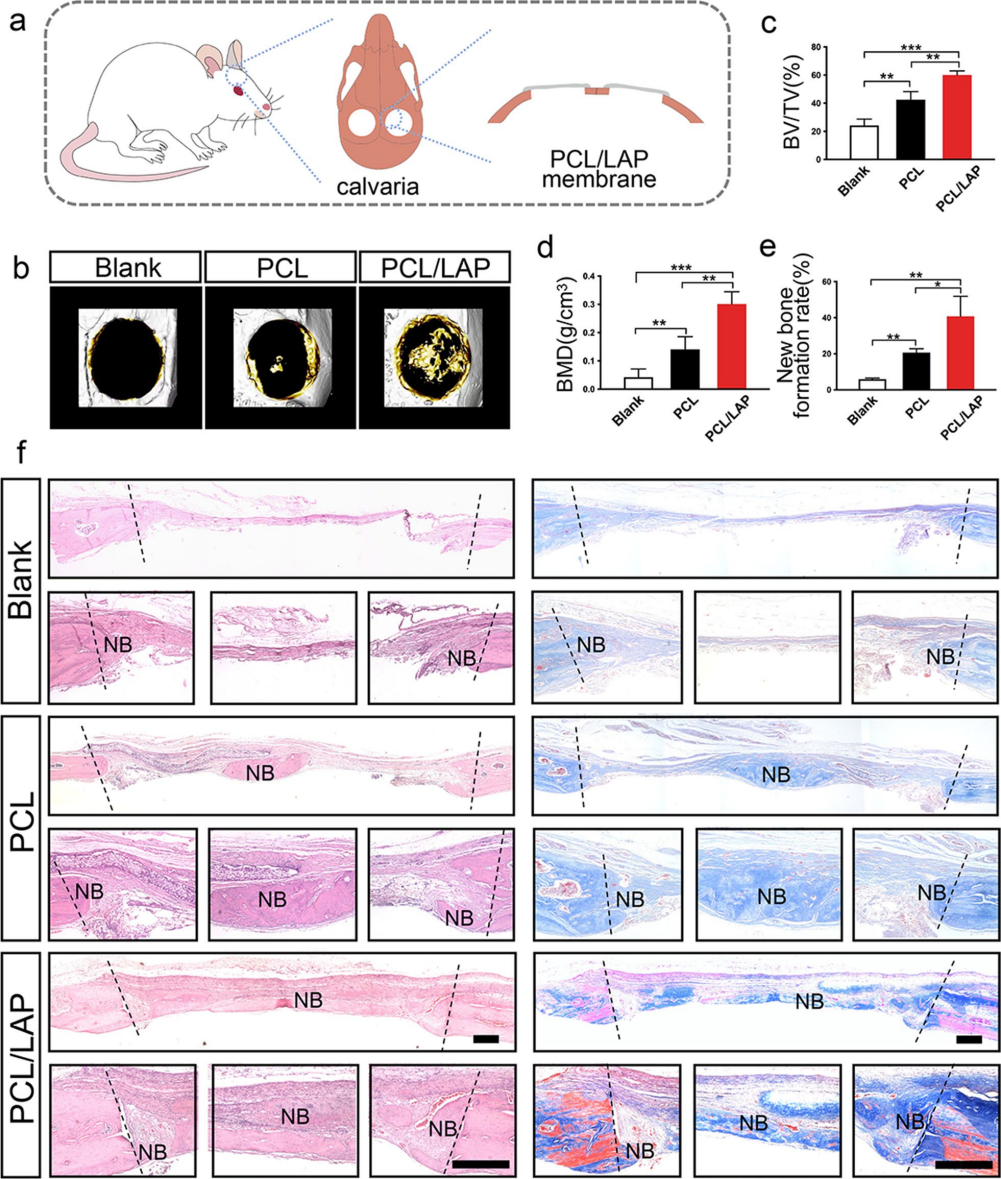
PCL/LAP nanofibrous membrane accelerates rat calvarial bone formation. **a** Schematic illustration of rat calvarial bone defect treated with PCL/LAP nanofibrous membrane. **b** Representative micro-CT reconstructed images of rat calvarial bone defects treated with PCL/LAP nanofibrous membranes. **c-d** BMD and BV/TV ratio of micro-CT data analysis. **e** Histological quantitative analysis of new bone formation rate. **f** Histological H&E- and Masson’s trichome-stained sections rat calvarial defects. NB, new bone. Scale bar = 200 μm. *P ≤ 0.05; **P ≤ 0.01; ***P ≤ 0.001

A rat calvarial bone defect was prepared and treated with PCL or PCL/LAP nanofibrous membrane (Fig. 7a). Representative three-dimensional images of rat calvarial bone after nanofibrous membranes implantation for 8 weeks (Fig. 7b). The micro-CT data analysis indicated that BMD and BV/TV ratio of new bone in the defects significantly increased in the PCL/LAP group (Fig. 7c-d).

Figure 7f show histological sections of rat calvarial defects after PCL/LAP nanofibrous membrane treatment. Only few new bone formed around the calvarial defect in the blank group. In the PCL group, limited new bone formation could be observed in the edge and center of defect. Abundant layer new bone formed and regenerated in the rat calvarial defects after PCL/LAP nanofibrous membrane implantation. Quantitative analysis of each group indicated that new bone formation areas were significantly higher in the PCL/LAP group than in the blank group and PCL group (Fig. 7e). The results indicated that both the PCL and PCL/LAP membranes promoted new bone formation compared with the blank group, and the highest new bone formation rate was found in the PCL/LAP group.

### PCL/LAP nanofibrous membrane promotes rat periodontal regeneration

**Fig. 8:**
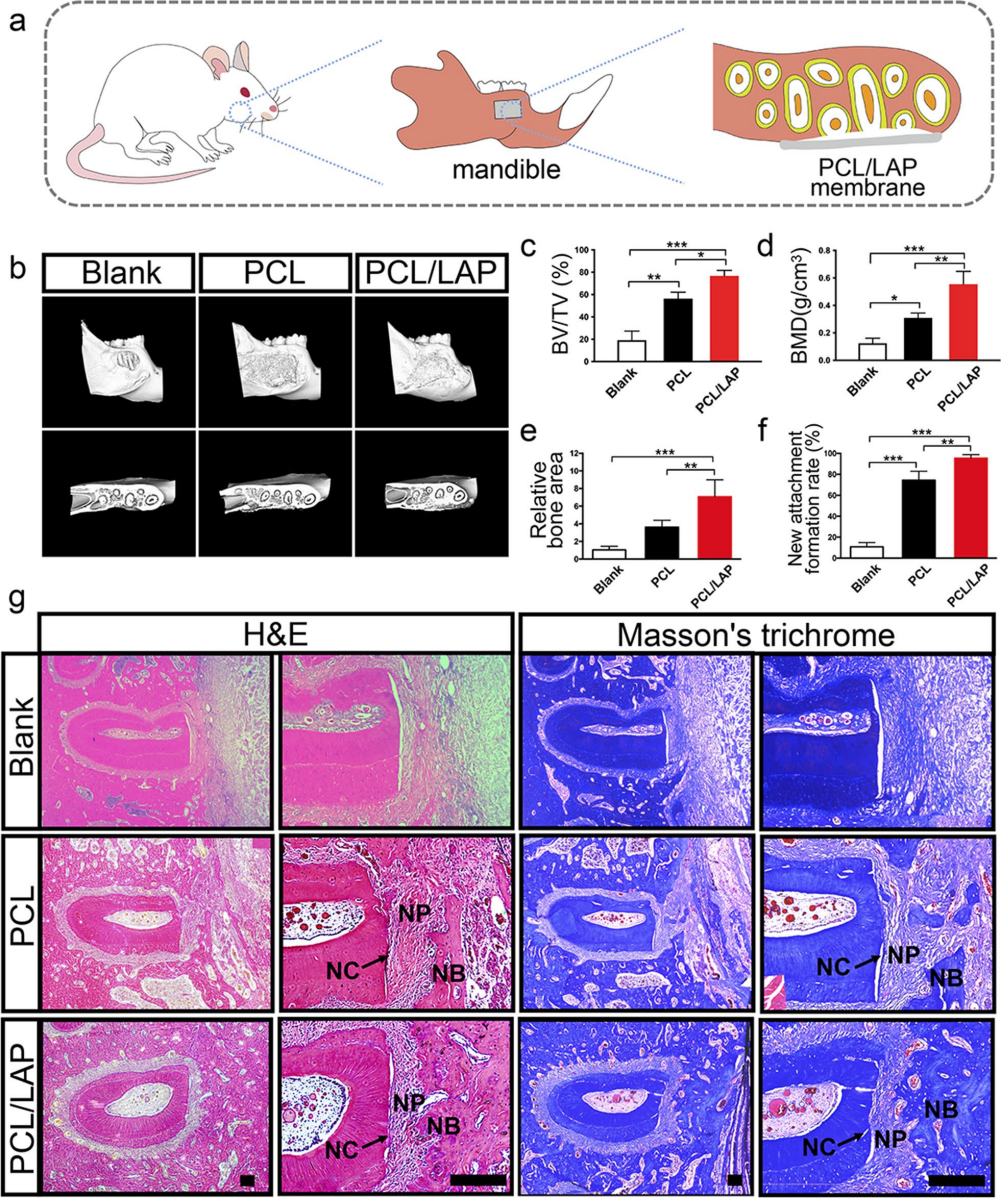
PCL/LAP nanofibrous membrane promotes rat mandibular periodontal defect regeneration. **a** Schematic illustration of rat periodontal defect treated with PCL/LAP nanofibrous membrane. **b** Representative micro-CT reconstructed images of periodontal defects treated with PCL/LAP nanofibrous membranes. **c-d** BV/TV ratio and BMD of Micro-CT data analysis. **e-f** Histological quantitative analysis of new bone area and new attachment formation rate. **g** Histological H&E- and Masson’s trichome-stained sections of rat periodontal defects. NC, new cementum; NP, new periodontal ligaments; NB, new bone. Scale bar = 200 μm. *P ≤ 0.05; **P ≤ 0.01; ***P ≤ 0.001

A rat periodontal defect was prepared and treated with PCL or PCL/LAP membrane (Fig. 8a). Representative three-dimensional images of each group showed that the PCL/LAP nanofibrous membrane could improve bone filling after four weeks (Fig. 8b). The micro-CT data were analyzed, and the results indicated that the BV/TV ratio and BMD of new bone in periodontal defects significantly increased in the PCL/LAP group (Fig. 8c, d).

Figure 8 g shows histological sections of rat periodontal defects after PCL/LAP nanofibrous membrane treatment. No obvious negative effect could be found in the defect, which confirmed the biocompatibility of the PCL/LAP nanofibrous membrane. Only disordered and unorganized fibers infiltrated the periodontal defect in the blank group without any implants at four weeks postsurgery. Limited bone regeneration occurred, and some collagen fibers connected to newly formed bone and root surfaces after PCL nanofibrous membrane implantation. Notably, dense and mature bone formed around the exposed root below the PCL/LAP nanofibrous membrane. Uniform collagen fibers adjacent to the surface of the root and mature bone to form new attachments in periodontal defects could be observed in the PCL/LAP group.

Quantitative analysis of each group indicated that new bone formation areas were significantly higher in the PCL/LAP group than in the blank group and PCL group (Fig. 8e). To further assess new attachment formation, the areas of collagen fibers inserted into new bone and the root surface were calculated. The results indicated that both the PCL and PCL/LAP membranes promoted new attachment formation compared with the blank group, and the highest new attachment formation rate was found in the PCL/LAP group (Fig. 8f).

## Discussion

An ideal biomaterial for periodontal regeneration should maintain a stable area and provide a pro-regenerative microenvironment `[3]. Accumulating studies have focused on biomaterial modification to enhance its pro-regenerative properties, including coating and incorporation of ions or growth factors [2, 25]. We previously developed LAP nanoparticle-incorporated PCL nanocomposites providing cumulative effects on osteogenesis, angiogenesis and osteoclastogenesis during bone regeneration [16, 17]. The PCL/LAP nanofibrous membrane in the present study was created to aid in periodontal regeneration by mobilizing remodeling constructively PDLCs to mobilize regeneration processes. Our results demonstrated, for the first time, that the PCL/LAP nanofibrous membrane improved proliferation, enhanced osteogenesis, attenuated the inflammatory response of neutrophils and macrophages, and induced regenerative N2 neutrophil and M2 macrophage polarization by exerting the immunomodulatory effects of PDLCs. Moreover, the PCL/LAP nanofibrous membrane facilitated rat calvarial bone formation and periodontal defect repairing in vivo. These results suggest that the PCL/LAP nanofibrous membrane is dynamically involved in a series of processes during periodontal regeneration and has the potential to orchestrate recruited PDLCs in periodontal defects to exert osteogenensis and immunomodulation for periodontal regeneration. This confirms its potential for application in periodontal regeneration therapy.

PCL is a widely used polymer with superior biocompatibility, and LAP is biocompatible and noncytotoxic within a suitable concentration range [11, 14, 15, 26]. A series of nanosilicate-incorporated PCLs with different amounts of LAP particles were developed in our previous study [16, 17], and we found that PCL with 5 wt% LAP had excellent biological properties. Therefore, the nanofibrous membrane with 5 wt% LAP incorporation in the present study was customized by electrospinning. Our results demonstrated PCL/LAP nanofibrous membrane possessed higher mechanical properties, which might contribute to the incorporation of LAP with a exfoliation status [17]. The PCL/LAP nanofibrous membrane had no adverse effects on PDLCs. Moreover, PDLCs cultured on the PCL/LAP nanofibrous membrane proliferated more rapidly, which confirmed the biocompatibility of the PCL/LAP nanofibrous membrane. These results were similar to studies of other LAP-containing biomaterials on bone marrow mesenchymal stem cells [27–30].

Osteogenesis plays a vital role in the entire periodontal regeneration process. Various periodontal regeneration strategies have been employed to improve the osteogenesis of PDLCs [31–33]. The current study demonstrated that the PCL/LAP nanofibrous membrane activated the expression of RUNX2, a key transcription factor during the osteogenic differentiation process. Its downstream targets (ALPL and COL1A1) increased accordingly, which ultimately improved mineral nodule formation in the late phase of osteogenesis. Our previous study indicated that LAP was released from the PCL/LAP nanocomposite and was internalized by cells to stimulate osteogenic differentiation [17]. Some studies also confirmed that LAP exerted extensive osteogenic effects in osteoblasts and bone mesenchymal cells [14, 15, 34–38]. Therefore, we speculated that LAP released from the membrane accounts for the increased osteogenesis of PDLCs.

An immune inflammatory response occurs during the early phase of periodontal regeneration [39, 40]. Increasing evidence demonstrated both neutrophils and macrophages in the circulating leukocytes are firstly recruited to infiltrate into defect sites to involve tissue regeneration [41–43]. Neutrophil has been recently proved to polarize into distinct phenotypes, including pro-inflammatory phenotype (referred to as N1 neutrophil) and anti-inflammatory and pro-remodeling phenotype (referred to as N2 neutrophil) [44, 45]. N2 neutrophils in regenerative site secrete distinct sets of cytokines, such as anti-inflammatory cytokines (IL-4 and IL-10) and pro-remodeling cytokine (VEGFA), which benefit tissue regeneration [45, 46]. Previous study indicated that PCL would induce slight foreign body reaction and mildly increase pro-inflammatory responses within physiological range [47–49]. The present study also demonstrated conditioned medium derived from PDLCs cultured on PCL increased inflammatory responses compared with conditioned medium derived from PDLCs cultured on well plates with special coatings for cells and closer to physiological condition. The distinct culture surface for PDLCs and slight foreign body reaction of PCL might be attributed to this effects. Multi-cellular interactions occur in periodontal defect after regeneration therapy. Therefore, a PDLCs and neutrophils coculture system was fabricated in the present study via a conditioned medium method to originally explored whether PDLCs cultured on PCL/LAP nanofibrous membrane would mediate neutrophil polarization. Under different conditioned media from PDLCs, neutrophils treated with conditioned media from PDLCs cultured on PCL/LAP nanofibrous membrane expressed higher N2 neutrophil-specific cytokine, which favor tissue regeneration.

The M1-to-M2 transition of macrophages is effective in mediating tissue regeneration [39, 50]. It has been reported that macrophage deficiency in the local niche seriously compromises tissue healing [51]. Moreover, PDLCs were capable of switching macrophages from M1 to M2 polarization in vitro [39]. PDLCs implanted into periodontal defects were able to manipulate inflammatory responses and induce M2 macrophage polarization to stimulate periodontal regeneration [20]. Therefore, PDLC and macrophage were co-cultured in the current study via a conditioned medium method. PDLCs cultured on the PCL/LAP membrane reduced proinflammatory responses and induced M2 macrophage polarization to endow favorable immunomodulation. Very few studies have focused on the effect of LAP on macrophages. Li et al. reported that RAW 264.7 macrophages treated with LAP secrete higher proinflammatory cytokines and polarize into M1 macrophages [26]. Hence, the anti-inflammatory effects might contribute to immunomodulation of PDLCs cultured on PCL/LAP nanofibrous membranes. To the best of our knowledge, this is the first study to reveal the immunomodulatory effect of PDLCs orchestrated by LAP.

Alveolar bone is the dominant components of periodontal tissue. Alveolar bone regeneration is the prerequisite and basis for achieving structural and functional regeneration of periodontal tissue [1]. The current study implanted PCL/LAP nanofibrous membrane into rat calvarial defect and found more bone formation. It confirmed the pro-regenerative property of PCL/LAP nanofibrous membrane in new bone regeneration. Ideal periodontal regeneration not only requires bone, pe riodontal ligament fibers and cementum formation, but also demands the periodontal ligament fibers inserting into newly formed bone and cementum of root surface to reconstrued functional periodontal tissue [52]. To evaluate the effect of PCL/LAP nanofibrous membrane on periodontal regeneration, a rat mandible fenestration model is recognized as a common periodontal defect model and has been widely used in various studies [53]. We previously investigated the potential of leptin gene therapy on periodontal regeneration in such a periodontal defect model [24]. In the current study, the PCL/LAP nanofibrous membrane was placed in the periodontal defect model to explore the effect of the PCL/LAP fibrous membrane on periodontal regeneration. Our study indicated that the PCL/LAP nanofibrous membrane remains more effective in improving the regeneration of new bone to form attachments in periodontal defects. We speculated that the LAP released from the PCL/LAP membrane in periodontal defects might mobilize self-regeneration by directly promoting the osteogenesis of local PDLCs and indirectly mediating inflammatory responses and M2 macrophage polarization by tuning the immunomodulation of PDLCs, which eventually leads to periodontal regeneration.

Although the effects of the PCL/LAP nanofibrous membrane on periodontal regeneration have been evaluated, only PDLCs used in the present study are limited enough to elucidate the complex periodontal regeneration process involving a series of cellular events. Importantly, the inflammatory responses and polarization of neutrophils and macrophages have been demonstrated to be time-dependent during tissue regeneration [50, 54], but they were not included in the current study. The evidence of the immunomodulatory effect of the PCL/LAP nanofibrous membrane in vivo is absent due to neutrophil and macrophage-involved regeneration processes, mainly occurring in the early stage. In addition, the particular mechanisms of the effects of PCL/LAP nanofibrous membranes on PDLC-mediated periodontal regeneration remain unknown. These unknowns will offer new insights into the influences of PCL/LAP nanofibrous membrane-induced periodontal regeneration in our forthcoming studies.

## Conclusions

**Fig. 9:**
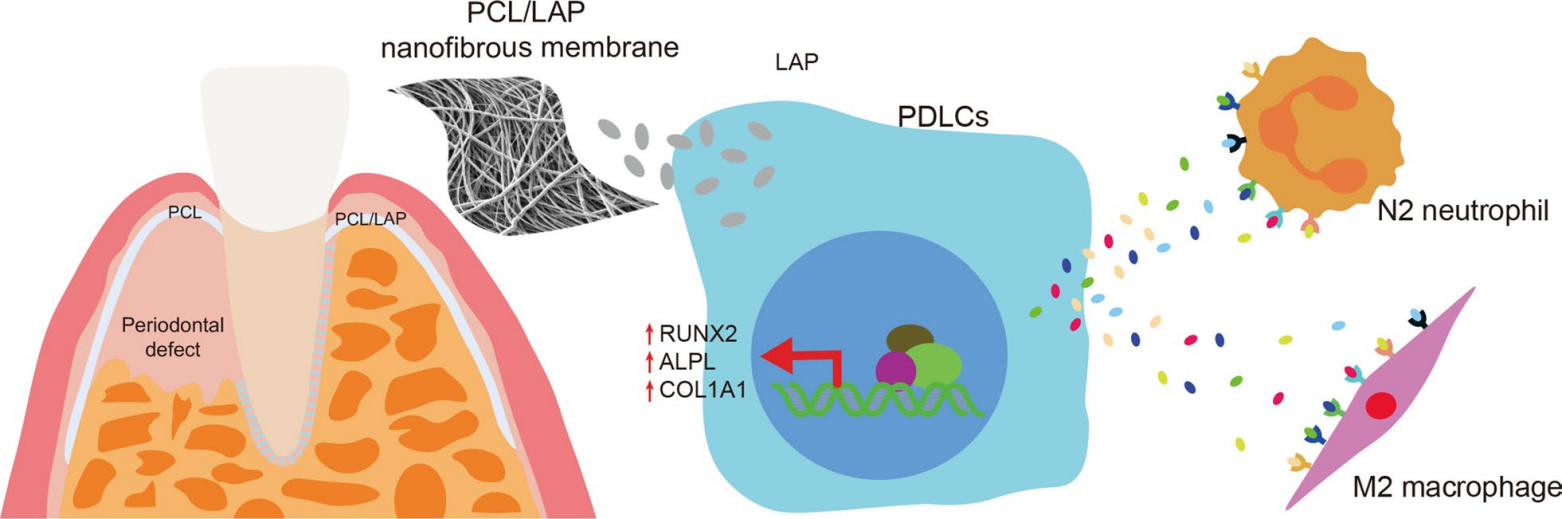
Graphical model of nanosilicate-functionalized PCL membrane improved periodontal regeneration by regulating PDLCs-mediated osteogenesis and immunomodulation in microenvironments after periodontal regeneration therapy

A nanosilicate-functionalized PCL membrane was successfully generated and evaluated to determine its potential utility in periodontal regeneration in the present study. It was biocompatible and stimulated the proliferation and osteogenesis of PDLCs. It also regulated inflammatory responses and induced N2 neutrophil and M2 macrophage polarization by orchestrating the immunomodulation of PDLCs. PCL/LAP implanted in vivo had a synergistic effect on rat periodontal tissue regeneration (Fig. 9). Our findings suggest that PCL/LAP is a promising biomaterial for periodontal regeneration therapy.

## References

[1] Slots J (2017). Periodontitis: facts, fallacies and the future. Periodontol 2000.

[2] Florjanski W, Orzeszek S, Olchowy A, Grychowska N, Wieckiewicz W, Malysa A, Smardz J, Wieckiewicz M (2019). Modifications of polymeric membranes used in guided tissue and bone regeneration. Polymers.

[3] Omar O, Elgali I, Dahlin C, Thomsen P (2019). Barrier membranes: more than the barrier effect?. J Clin Periodontol.

[4] Li J, Chen M, Wei X, Hao Y, Wang J (2017). Evaluation of 3D-Printed polycaprolactone scaffolds coated with freeze-dried platelet-rich plasma for bone regeneration. Mater.

[5] Zhang L, Dong Y, Zhang N, Shi J, Zhang X, Qi C, Midgley A, Wang S (2020). Potentials of sandwich-like chitosan/polycaprolactone/gelatin scaffolds for guided tissue regeneration membrane. Mater Sci Eng C Mater Biol Appl.

[6] Sunandhakumari V, Vidhyadharan A, Alim A, Kumar D, Ravindran J, Krishna A, Prasad M (2018). Fabrication and in vitro characterization of bioactive glass/nano hydroxyapatite reinforced electrospun poly(ε-Caprolactone) composite membranes for guided tissue regeneration. Bioeng.

[7] Farag A, Hashimi S, Vaquette C, Bartold P, Hutmacher D, Ivanovski S (2018). The effect of decellularized tissue engineered constructs on periodontal regeneration. J Clin Periodontol.

[8] Farag A, Hashimi S, Vaquette C, Volpato F, Hutmacher D, Ivanovski S (2018). Assessment of static and perfusion methods for decellularization of PCL membrane-supported periodontal ligament cell sheet constructs. Arch Oral Biol.

[9] Hasani-Sadrabadi M, Sarrion P, Nakatsuka N, Young T, Taghdiri N, Ansari S, Aghaloo T, Li S, Khademhosseini A, Weiss P, Moshaverinia A (2019). Hierarchically patterned polydopamine-containing membranes for periodontal tissue engineering. ACS Nano.

[10] Pilipchuk S, Monje A, Jiao Y, Hao J, Kruger L, Flanagan C, Hollister S, Giannobile W (2016). Integration of 3D printed and Micropatterned Polycaprolactone Scaffolds for Guidance of oriented collagenous tissue formation in vivo. Adv Healthc Mater.

[11] Gaharwar A, Cross L, Peak C, Gold K, Carrow J, Brokesh A, Singh K (2019). 2D nanoclay for biomedical applications: regenerative medicine, therapeutic delivery, and additive manufacturing. Adv Mater.

[12] Kiaee G, Dimitrakakis N, Sharifzadeh S, Kim H, Avery R, Moghaddam K, Haghniaz R, Yalcintas E, Barros N, Karamikamkar S, Libanori A, Khademhosseini A, Khoshakhlagh P (2022). Laponite-Based nanomaterials for drug delivery. Adv Healthc Mater.

[13] Mohanty RP, Joshi YM (2014). Chemical stability of Laponite in aqueous media. Appl Clay Sci.

[14] Carrow J, Cross L, Reese R, Jaiswal M, Gregory C, Kaunas R, Singh I, Gaharwar A (2018). Widespread changes in transcriptome profile of human mesenchymal stem cells induced by two-dimensional nanosilicates. Proc Natl Acad Sci USA.

[15] Mousa M, Milan J, Kelly O, Doyle J, Evans N, Oreffo R, Dawson J (2021). The role of lithium in the osteogenic bioactivity of clay nanoparticles. Biomater Sci.

[16] Xu X, Xiao L, Xu Y, Zhuo J, Yang X, Li L, Xiao N, Tao J, Zhong Q, Li Y, Chen Y, Du Z, Luo K (2021). Vascularized bone regeneration accelerated by 3D-printed nanosilicate-functionalized polycaprolactone scaffold. Regener Biomater.

[17] Xu X, Zhuo J, Xiao L, Xu Y, Yang X, Li Y, Du Z, Luo K (2022). Nanosilicate-Functionalized polycaprolactone orchestrates osteogenesis and osteoblast-Induced multicellular interactions for potential endogenous vascularized bone regeneration. Macromol Biosci.

[18] Xiong J, Gronthos S, Bartold P (2013). Role of the epithelial cell rests of Malassez in the development, maintenance and regeneration of periodontal ligament tissues. Periodontol 2000.

[19] Iwata T, Yamato M, Zhang Z, Mukobata S, Washio K, Ando T, Feijen J, Okano T, Ishikawa I (2010). Validation of human periodontal ligament-derived cells as a reliable source for cytotherapeutic use. J Clin Periodontol.

[20] Liu J, Chen B, Bao J, Zhang Y, Lei L, Yan F (2019). Macrophage polarization in periodontal ligament stem cells enhanced periodontal regeneration. Stem Cell Res Ther.

[21] Liu J, Wang H, Zhang L, Li X, Ding X, Ding G, Wei F (2022). Periodontal ligament stem cells promote polarization of M2 macrophages. J Leukoc Biol.

[22] Konermann A, Beyer M, Deschner J, Allam JP, Novak N, Winter J, Jepsen S, Jäger A (2012). Human periodontal ligament cells facilitate leukocyte recruitment and are influenced in their immunomodulatory function by Th17 cytokine release. Cell Immunol.

[23] Haase H, Ivanovski S, Waters M, Bartold P (2003). Growth hormone regulates osteogenic marker mRNA expression in human periodontal fibroblasts and alveolar bone-derived cells. J Periodontal Res.

[24] Zheng B, Jiang J, Chen Y, Lin M, Du Z, Xiao Y, Luo K, Yan F (2017). Leptin overexpression in bone marrow stromal cells promotes Periodontal Regeneration in a rat model of osteoporosis. J Periodontol.

[25] Wang J, Wang L, Zhou Z, Lai H (2022). Biodegradable polymer membranes applied in guided bone/tissue regeneration: a review. Polymers.

[26] Li T, Liu Z, Xiao M, Yang Z, Peng M, Li C, Zhou X, Wang J (2018). Impact of bone marrow mesenchymal stem cell immunomodulation on the osteogenic effects of laponite. Stem Cell Res Ther.

[27] Topuz F, Nadernezhad A, Caliskan O, Menceloglu Y, Koc B (2018). Nanosilicate embedded agarose hydrogels with improved bioactivity. Carbohydr Polym.

[28] Page DJ, Clarkin CE, Raj M (2019). Injectable nanoclay gels for angiogenesis. Acta Biomater.

[29] Okesola BO, Ni S, Derkus B, Galeano CC, Hasan A, Wu Y, Ramis J, Buttery L, Dawson JI, D’Este M, Oreffo ROC, Eglin D, Sun H, Mata A (2020). Growth-factor free multicomponent nanocomposite hydrogels that stimulate bone formation. Adv Funct Mater.

[30] Dong Q, Cai J, Wang H, Chen S, Liu Y, Yao J, Shao Z, Chen X (2020). Artificial ligament made from silk protein/Laponite hybrid fibers. Acta Biomater.

[31] Woo H, Cho Y, Tarafder S, Lee C (2021). The recent advances in scaffolds for integrated periodontal regeneration. Bioact Mater.

[32] Huang Q, Huang X, Gu L (2021). Periodontal bifunctional biomaterials: progress and perspectives. Mater.

[33] Park C (2019). Biomaterial-Based approaches for regeneration of periodontal ligament and cementum using 3D platforms. Int J Mol Sci.

[34] Ibrahim D, Sani E, Soliman A, Zandi N, Mostafavi E, Youssef A, Allam N, Annabi N (2020). Bioactive and elastic nanocomposites with antimicrobial properties for bone tissue regeneration. ACS Appl Bio Mater.

[35] Liu Z, Shang L, Ge S (2021). Immunomodulatory effect of dimethyloxallyl glycine/nanosilicates-loaded fibrous structure on periodontal bone remodeling. J Dent Sci.

[36] Zhang X, Fan J, Lee C, Kim S, Chen C, Lee M (2020). Supramolecular hydrogels based on nanoclay and guanidine-rich chitosan: injectable and moldable osteoinductive carriers. ACS Appl Mater Interfaces.

[37] Cidonio G, Cooke M, Glinka M, Dawson J, Grover L, Oreffo R (2019). Printing bone in a gel: using nanocomposite bioink to print functionalised bone scaffolds. Mater Today Bio.

[38] Zheng X, Zhang X, Wang Y, Liu Y, Pan Y, Li Y, Ji M, Zhao X, Huang S, Yao Q (2021). Hypoxia-mimicking 3D bioglass-nanoclay scaffolds promote endogenous bone regeneration. Bioact Mater.

[39] Li X, He X, Kong D, Xu X, Wu R, Sun L, Tian B, Chen F (2019). M2 macrophages enhance the Cementoblastic differentiation of Periodontal ligament stem cells via the akt and JNK pathways. Stem Cells.

[40] Ni C, Zhou J, Kong N, Bian T, Zhang Y, Huang X, Xiao Y, Yang W, Yan F (2019). Gold nanoparticles modulate the crosstalk between macrophages and periodontal ligament cells for periodontitis treatment. Biomaterials.

[41] Forbes S, Rosenthal N (2014). Preparing the ground for tissue regeneration: from mechanism to therapy. Nat Med.

[42] Medeiros N, Mattos R, Menezes C, Fares R, Talvani A, Dutra W, Rios-Santos F, Correa-Oliveira R, Gomes J (2018). IL-10 and TGF-β unbalanced levels in neutrophils contribute to increase inflammatory cytokine expression in childhood obesity. Eur J Nutr.

[43] Sorkin M, Huber A, Hwang C, Carson W, Menon R, Li J, Vasquez K, Pagani C, Patel N, Li S, Visser N, Niknafs Y, Loder S, Scola M, Nycz D, Gallagher K, McCauley L, Xu J, James A, Agarwal S, Kunkel S, Mishina Y, Levi B (2020). Regulation of heterotopic ossification by monocytes in a mouse model of aberrant wound healing. Nat Commun.

[44] Lin R, Lee C, Moreno-Luna R, Neumeyer J, Piekarski B, Zhou P, Moses M, Sachdev M, Pu W, Emani S, Melero-Martin J (2017). Host non-inflammatory neutrophils mediate the engraftment of bioengineered vascular networks. Nat Biomed Eng.

[45] Cai B, Lin D, Li Y, Wang L, Xie J, Dai T, Liu F, Tang M, Tian L, Yuan Y, Kong L, Shen S (2021). N2-Polarized Neutrophils Guide Bone mesenchymal stem cell recruitment and initiate bone regeneration: a missing piece of the bone regeneration puzzle. Adv Sci.

[46] Luz-Crawford P, Kurte M, Bravo-Alegría J, Contreras R, Nova-Lamperti E, Tejedor G, Noël D, Jorgensen C, Figueroa F, Djouad F, Carrión F (2013). Mesenchymal stem cells generate a CD4 + CD25 + Foxp3 + regulatory T cell population during the differentiation process of Th1 and Th17 cells. Stem Cell Res Ther.

[47] Sandri S, Hebeda CB, Loiola RA, Calgaroto S, Uchiyama MK, Araki K, Frank LA, Paese K, Guterres SS, Pohlmann AR, Farsky SHP (2019). Direct effects of poly(ε-caprolactone) lipid-core nanocapsules on human immune cells. Nanomed.

[48] Dong C, Qiao F, Chen G, Lv Y (2021). Demineralized and decellularized bone extracellular matrix-incorporated electrospun nanofibrous scaffold for bone regeneration. J Mater Chem B.

[49] Wang Z, Cui Y, Wang J, Yang X, Wu Y, Wang K, Gao X, Li D, Li Y, Zheng XL, Zhu Y, Kong D, Zhao Q (2014). The effect of thick fibers and large pores of electrospun poly(ε-caprolactone) vascular grafts on macrophage polarization and arterial regeneration. Biomaterials.

[50] Qiu P, Li M, Chen K, Fang B, Chen P, Tang Z, Lin X, Fan S (2020). Periosteal matrix-derived hydrogel promotes bone repair through an early immune regulation coupled with enhanced angio- and osteogenesis. Biomaterials.

[51] Németh K, Leelahavanichkul A, Yuen P, Mayer B, Parmelee A, Doi K, Robey P, Leelahavanichkul K, Koller B, Brown J, Hu X, Jelinek I, Star R, Mezey E (2009). Bone marrow stromal cells attenuate sepsis via prostaglandin E(2)-dependent reprogramming of host macrophages to increase their interleukin-10 production. Nat Med.

[52] Tassi S, Sergio N, Misawa M, Villar C (2017). Efficacy of stem cells on periodontal regeneration: systematic review of pre-clinical studies. J Periodontal Res.

[53] Padial-Molina M, Rodriguez J, Volk S, Rios H (2015). Standardized in vivo model for studying novel regenerative approaches for multitissue bone-ligament interfaces. Nat Protoc.

[54] Yin Y, He X, Wang J, Wu R, Xu X, Hong Y, Tian B, Chen F (2020). Pore size-mediated macrophage M1-to-M2 transition influences new vessel formation within the compartment of a scaffold. Appl Mater Today.

